# Ultrahigh efficient spin orbit torque magnetization switching in fully sputtered topological insulator and ferromagnet multilayers

**DOI:** 10.1038/s41598-022-06779-3

**Published:** 2022-02-22

**Authors:** Tuo Fan, Nguyen Huynh Duy Khang, Soichiro Nakano, Pham Nam Hai

**Affiliations:** 1 Department of Electrical and Electronic Engineering, Tokyo Institute of Technology, 2-12-1 Ookayama, Meguro, Tokyo 152-8550 Japan; 2 Department of Physics, Ho Chi Minh City University of Education, 280 An Duong Vuong Street, District 5, Ho Chi Minh City, 738242 Vietnam; 3 Center for Spintronics Research Network (CSRN), The University of Tokyo, 7-3-1 Hongo, Bunkyo, Tokyo 113-8656 Japan; 4 CREST, Japan Science and Technology Agency, 4-1-8 Honcho, Kawaguchi, Saitama 332-0012 Japan

**Keywords:** Nanoscience and technology, Materials science, Condensed-matter physics, Materials for devices

## Abstract

Spin orbit torque (SOT) magnetization switching of ferromagnets with large perpendicular magnetic anisotropy has a great potential for the next generation non-volatile magnetoresistive random-access memory (MRAM). It requires a high performance pure spin current source with a large spin Hall angle and high electrical conductivity, which can be fabricated by a mass production technique. In this work, we demonstrate ultrahigh efficient and robust SOT magnetization switching in fully sputtered BiSb topological insulator and perpendicularly magnetized Co/Pt multilayers. Despite fabricated by the magnetron sputtering instead of the laboratory molecular beam epitaxy, the topological insulator layer, BiSb, shows a large spin Hall angle of *θ*_SH_ = 10.7 and high electrical conductivity of *σ* = 1.5 × 10^5^ Ω^−1^ m^−1^. Our results demonstrate the feasibility of BiSb topological insulator for implementation of ultralow power SOT-MRAM and other SOT-based spintronic devices.

## Introduction

Embedded non-volatile memories have great impacts on energy-efficient electronics, including Internet-of-Thing, Artificially Intelligent (AI), among others. To be successful, non-volatile memories have to satisfy several requirements, such as high writing endurance, high capacity, high speed, and low fabrication cost. Among many emerging non-volatile memory technologies, magnetoresistive random-access memory (MRAM) is one of the most promising that have gained development commitment from several leading semiconductor companies. The latest MRAM technology using the sophisticated spin–transfer–torque (STT) writing technique has just been commercially available very recently, but already found various important applications, such as highly efficient AI chips. However, in STT-MRAM, a large writing current has to be injected directly to magnetic tunneling junctions (MTJs), which leads to reliability issues such as accelerated aging of the oxide tunnel barrier^1^. In addition, large writing currents require large driving transistors, making it difficult to increase the bit density of STT-MRAM beyond the 1 Gbit capacity. Recently, the spin–orbit–torque (SOT) technique has emerged as a promising writing method for the next generation MRAM^2^. In SOT-MRAM, a charge current flowing in a non-magnetic layer with large spin–orbit interaction can generate a pure spin current by the spin Hall effect. The pure spin current is then injected to the magnetic free layer for magnetization switching. The relationships between the spin current *I*_S_ and the charge current *I*_C_ is given by *I*_S_ = (ℏ/2*e*)(*L*/*t*)*θ*_SH_*I*_C_, where *L* is the MTJ size, and *t* the thickness of the spin Hall layer, and *θ*_SH_ is the spin Hall angle. Theoretically, the charge-to-spin conversion efficiency (*L*/*t*)*θ*_SH_ in SOT-MRAM can be larger than unity, meaning that lower driving currents can be expected. Furthermore, since there is no current flowing into the MTJs, reliability can be significantly improved. Finally, since the spin-polarization of the pure spin current is perpendicular to the magnetization direction of the free magnetic layer, the spin torque is maximized and the magnetization can switch very fast (< ns) in SOT-MRAM with perpendicular magnetic anisotropy (PMA)^3^. Because of those merits of SOT-MRAM comparing with STT-MRAM, there have been huge efforts to find spin Hall materials with large *θ*_SH_ and high electrical conductivity for SOT-MRAM implementation. Heavy metals, such as Pt^4,5^, Ta^2^, and W^6^, have been studied extensively as candidates for the spin Hall layer in SOT-MRAM, since they have been used in STT-MRAM manufacturing. However, *θ*_SH_ of heavy metals is of the order of ~ 0.1, and the typical critical switching current density in heavy metals/ferromagnet bilayers with PMA is of the order of 10^7^ Acm^−2^ to 10^8^ Acm^−2^, which is too high for Si electronics^7^. Thus, finding a material with large *θ*_SH_ of the order of 10 is of emergent need.

Recently, very large *θ*_SH_ (> 1) has been observed in topological insulators (TIs), making them very promising for SOT-MRAM^8,9^. TIs are quantum materials having gaped bulk states but Dirac-like metallic surface states^10–12^ with spin-momentum locking^13,14^, owning to their large spin–orbit interaction and band structure topology. Although the origin of large *θ*_SH_ of TIs was a controversial topic due to the coexistence of the bulk current and surface current^15^, it has been clearly shown to originate from the surface states rather than the bulk states in carefully controlled experiments^16^. However, in most TI thin films, the limited surface density of states restricts the electrical conductivity *σ* to the order of ~ 10^4^ Ω^−1^ m^−1^ (for example, *σ* ~ 5.7 × 10^4^ Ω^−1^ m^−1^ for Bi_2_Se_3_ and 1.8 × 10^4^ Ω^−1^ m^−1^ for (Bi_0.07_Sb_0.93_)_2_Te_3_), which results in a large shunting current when in contact with other metallic layers. Second, most TI thin films studied so far are single crystalline films grown epitaxially by the laboratory molecular beam epitaxy (MBE) technique on dedicated III-V semiconductor substrates, which is not compatible with mass production. Furthermore, ultralow power SOT magnetization switching at room-temperature with current densities of the order of 10^5^−10^6^ Acm^−2^ have been demonstrated mostly in TI/magnetic layers whose magnetic anisotropy is very small, such as in a Bi_2_Se_3_/NiFe bilayer with nearly zero in-plane magnetic anisotropy^17^, or in a Bi_2_Se_3_/CoTb bilayer with a small magnetization of 200 emu/cc and a PMA field of less than 2 kOe^18^. For TI being a practical material for SOT-MRAM, the following three minimum requirements must be satisfied: (1) a large spin Hall angle of the order of 10, (2) large electrical conductivity *σ* of order of 10^5^ Ω^−1^ m^−1^, and (3) can be deposited using sputtering deposition. The first two requirements are for optimization of the switching current density and switching energy (see Suppl. Info. Section 1). The closest attempt is Bi_x_Se_1-x_ thin films deposited by the sputtering technique, which shows a promisingly large *θ*_SH_ = 8.7–18.6 but with the expense of reduced electrical conductivity (*σ* ~ 7.8 × 10^3^ Ω^−1^ m^−1^)^19^. Thus, the three requirements for a practical spin Hall material for SOT-MRAM have not yet been achieved.

Among various TI material candidates, Bi_1-*x*_Sb_*x*_ (0.07 < *x* < 0.22) is the best hope. BiSb is the first discovered three-dimensional TI with small bulk bandgap (~ 20 meV) and high bulk conductivity *σ* of 4−6.4 × 10^5^ Ω^−1^ m^−1^^13,14,20^. In thin films, the quantum size effect significantly increases the band gap of BiSb, so that the current flows mostly on the surface when the thickness reaches 10 nm^21^. Thanks to the multi-surface states, *σ* of BiSb thin films is as high as 2.5 × 10^5^ Ω^−1^ m^−1^. Furthermore, a giant spin Hall effect with *θ*_SH_ ~ 52 has been observed in BiSb(012) thin films in junctions with MnGa grown on GaAs substrates by MBE^22^. Nevertheless, following works on sputtered polycrystalline BiSb yields a maximum *θ*_SH_ of only 1.2 ~ 2.4 (Refs.^23,24^), or even no spin Hall effect^25^. Therefore, it is very important to demonstrate the three requirements for sputtered BiSb for any realistic applications to SOT-based spintronic devices.

In this work, we demonstrate ultrahigh efficient SOT magnetization switching in fullly sputtered BiSb–(Co/Pt) multilayers with large PMA. We show that the sputtered BiSb has a large spin Hall angle of *θ*_SH_ = 10.7 and high electrical conductivity of *σ* = 1.5 × 10^5^ Ω^−1^ m^−1^, thus satisfying all the three requirements for SOT-MRAM implementation. Despite the large PMA field of 5.2 kOe of the (Co/Pt) multilayers, we achieve robust SOT magnetization at a low current density of 1.5 × 10^6^ Acm^−2^. Our results demonstrate the potential of BiSb topological insulator for ultralow power SOT-MRAM and other SOT-based spintronic devices.

## High quality topological insulator—perpendicularly magnetized ferromagnetic multilayers

The first step in this study is to demonstrate that it is possible to use the sputtering deposition technique for fabrication of high-quality topological insulator and perpendicularly magnetized ferromagnetic multilayers. Considering the large surface roughness of TIs (~ 5–6 Angstrom for BiSb) and atomic interdiffusion during the annealing process of the MTJ, it is not realistic to deposit the MTJ with large PMA on top of the TI layer. Instead, the MTJ should be deposited and fabricated first, then the TI layer should be deposited on top of the free magnetic layer at the last step. Furthermore, to achieve high enough thermal stability, the free magnetic layer should be composed of ferromagnetic multilayers with high PMA that couple ferromagnetically or antiferromagnetically to the CoFeB layer^26^. Noting that the (Co/Pt)_n_ (n = 2–6) multilayers have been frequently used in MRAM production as a part of the synthetic antiferromagnetic reference layer with large PMA for pinning, we chose the (Co 0.4 nm /Pt 0.4 nm)_2_ multilayers (referred below as CoPt) to realize thin ferromagnetic multilayers with large PMA for evaluating the SOT performance of the BiSb layer (see Suppl. Info. Section 2).

**Figure 1:**
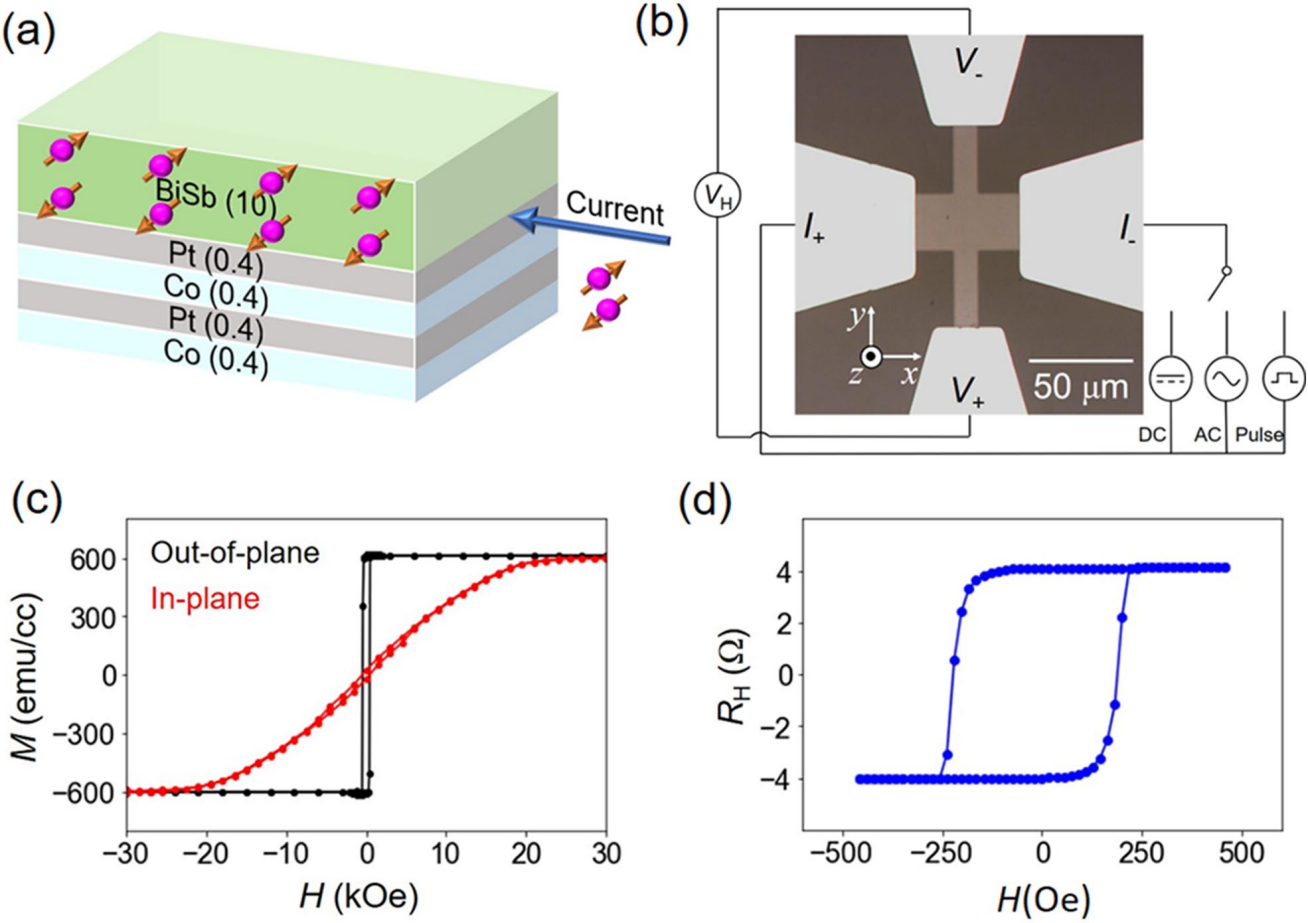
Perpendicularly magnetized Co/Pt ferromagnetic multilayers – topological insulator BiSb heterostructure. (**a**) Schematic structure of our multilayers. (**b**) Optical image of a Hall bar device and measurement configuration. (**c**) Magnetization curves of the Co/Pt multilayers. (**d**) Hall resistance of a Hall bar device measured with a perpendicular magnetic field.

Figure 1a shows the studied multilayer heterostructure, which consists of perpendicularly magnetized (Co 0.4 nm/Pt 0.4 nm)_2_ multilayers/10 nm Bi_0.8_Sb_0.15_ topological insulator layer, capped by 1 nm MgO/1 nm Pt (not shown). The multilayers were deposited on *c*-plane sapphire substrates by a combination of direct current (DC) and radio-frequency (RF) magnetron sputtering in a multi-cathode chamber. Characterization by X-ray diffraction and transmission electron microscopy shows that the BiSb layer is highly textured with the dominant (110) orientation (see Suppl. Info. Section 3). We also evaluated several sputtered BiSb single layers on sapphire substrates and observed the existence of metallic surface states as well as insulating bulk states with a band gap more than 170 meV for thin (< 20 nm) BiSb films^27^.

For electrical measurements, we fabricated 25 μm-wide Hall bars by optical lithography. An optical image of a Hall bar device with the experiment setup is shown in Fig. 1b. From the parallel resistor model, we estimate that the conductivity of the BiSb layer is 1.5 × 10^5^ Ω^−1^ m^−1^, which is much higher than that of sputtered Bi_1-*x*_Se_*x*_, and close to that of MBE grown BiSb on GaAs(111)A substrates. This demonstrates that is possible to grow highly conductive BiSb topological thin films on top of perpendicularly magnetized metallic layers by the sputtering technique. Thanks to the high conductivity of the BiSb layer, 50% of the applied current flows into the BiSb and contribute to the SOT magnetization switching.

Figure 1c shows the magnetic hysteresis curves of the as-grown sample measured by a superconducting quantum interference device (SQUID) under an in-plane and out-of-plane external magnetic field, respectively. The saturation magnetization *M*_CoPt_, normalised by the CoPt layer thickness (*t*_CoPt_ = 1.6 nm), is 613 emu⋅cm^−3^. The uniaxial anisotropy field *H*_k_ = 15 kOe is very large for the as-grown film. After Hall bar device fabrication by optical lithography undergoing several circles of thermal annealing, *H*_k_ is reduced to 5.2 kOe (see Suppl. Info. Section 4), but is still much larger than that of NiFe or CoTb used in previous works. This CoPt layer yields a thermal stability factor for magnetization switching of Δ = 38, similar to that of the CoFeB(/MgO) free layer of perpendicular MTJs with diameters of 20–40 nm^28,29^. Figure 1d shows the anomalous Hall resistance (*R*_H_) measured for a Hall bar under a sweeping out-of-plane field, which confirms PMA of the CoPt layer.

## Evaluation of the spin Hall angle by the second harmonic Hall measurements

**Figure 2:**
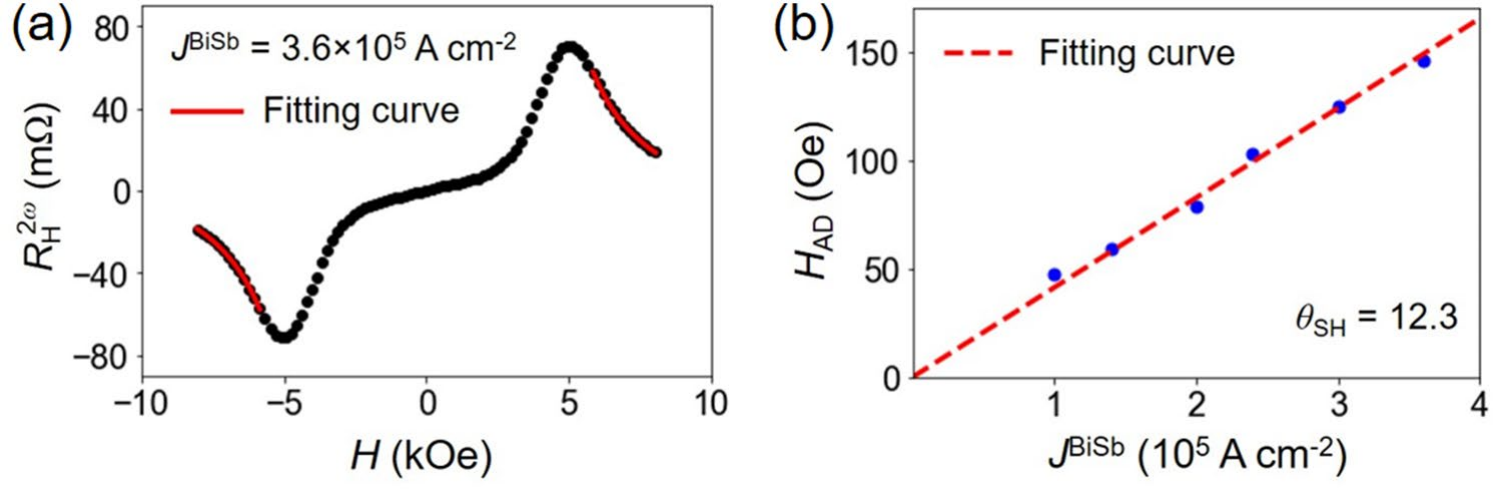
Evaluation of the spin Hall angle by the second harmonic measurements. (**a**) 2nd harmonic Hall resistance as a function of the in-plane external magnetic field *H* applied along the *x* direction. The red curves are the theoretical fitting using Eq. (1). (**b**) *H*_AD_ as a function of *J*^BiSb^.

Next, we performed the second harmonic Hall measurements to evaluate the spin Hall angle^30^. An alternating current (AC) *J* = *J*_0_sin *ω*t (*ω* = 259.68 Hz) was applied to the Hall bar under a sweeping external field along the *x* direction. We measured the 2nd harmonic Hall resistance $R_{\text{H}}^{{2{\upomega }}}$, which is originated from the oscillation of the net magnetic moment under the spin–orbit effective magnetic fields^31–33^. Figure 2a shows a representative $R_{\text{H}}^{{2{\upomega }}}$- *H*_*x*_ curve measured at *J*^BiSb^ = 3.6 × 10^5^ Acm^−2^. The second harmonic Hall resistance $R_{\text{H}}^{{2{\upomega }}}$ can be expressed as^33^

1 $$R_{\text{H}}^{{2{\upomega }}} = \frac{{R_{{\text{H}}} }}{{2}}\frac{{H_{{{\text{AD}}}} }}{{H_{x} - H_{{\text{k}}} (H_{x} /\left| {H_{x} } \right|)}} + R_{{{\text{PHE}}}} \frac{{H_{{{\text{FL}} + {\text{Oe}}}} }}{{H_{{x}} }} + R_{{{\text{thermal}}}} \frac{{H_{x} }}{{{|}H_{x} {|}}}$$

where *H*_AD_ is the antidamping-like effective field, *H*_FL+Oe_ is the sum of the fieldlike and Oesterd field, *R*_PHE_ is the planar Hall resistance, and *R*_thermal_ is the contribution from the anomalous Nernst (ANE) and spin Seebeck (SSE) effects. Note that the contribution of the fieldlike and Oesterd field to $R_{\text{H}}^{{2{\upomega }}}$ is much smaller than that of the antidampinglike field in BiSb^34,35^. Fitting Eq. (1) to the high field data in the $R_{\text{H}}^{{2{\upomega }}}$- *H*_*x*_ curve yields *H*_AD_ (red curves in Fig. 2a). Figure 2b shows *H*_AD_ as a function of *J*^BiSb^. From the *H*_AD_ / *J*^BiSb^ gradient, we can calculate the effective spin Hall angle ${\theta }_{\mathrm{SH}}^{\mathrm{eff}}$ = $\frac{2e}{\hbar }M_{{{\text{CoPt}}}} t_{{{\text{CoPt}}}} \frac{{H_{{{\text{AD}}}} }}{{J^{{{\text{BiSb}}}} }}$ = 12.3, where *e* is the electron charge and $\hbar$ is the reduced Plank constant. Recently, it was reported that CoPt multilayers can generate a “self” spin–orbit torque^36^. We found that the “self” spin–orbit torque in CoPt can contribute to 13% of the total spin–orbit torque (see Suppl. Info. Section 6). Furthermore, we confirmed that there is no artifact contribution from the asymmetric magnon scattering mechanism (see Suppl. Info. Section 7), as observed in the case of the magnetic topological insulator (CrBiSb)_2_Te_3_^37^. Subtracting the contribution from CoPt, we obtain the intrinsic spin Hall angle of BiSb *θ*_SH_ = 10.7, which demonstrates the feasibility of BiSb for ultralow power SOT-MRAM.

## Ultrahigh efficient spin–orbit torque magnetization switching by DC and pulse currents

**Figure 3:**
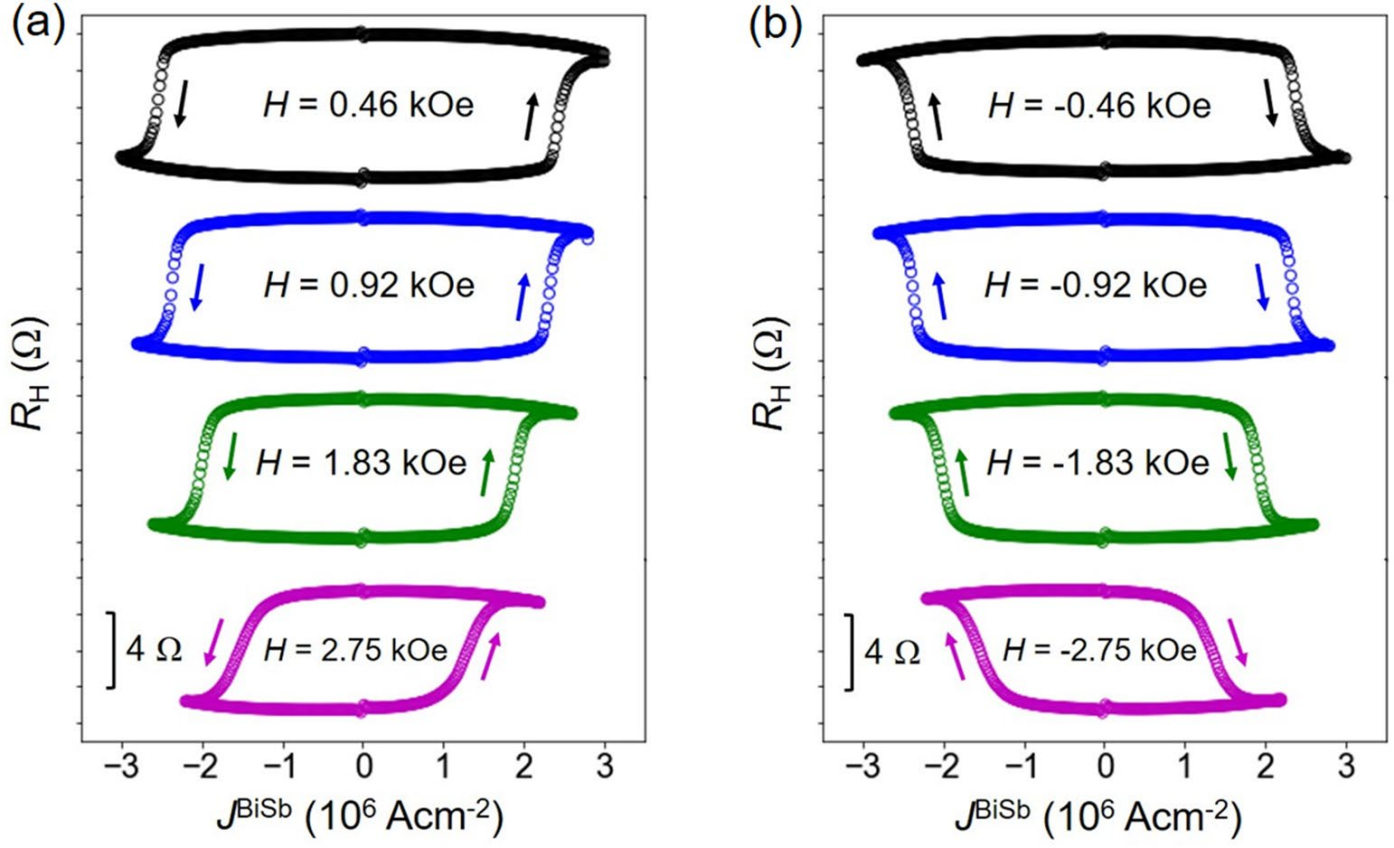
SOT magnetization switching by DC currents. Switching loops measured under an in-plane magnetic field applied along (**a**) + *x* direction and (**b**) − *x* direction.

**Figure 4:**
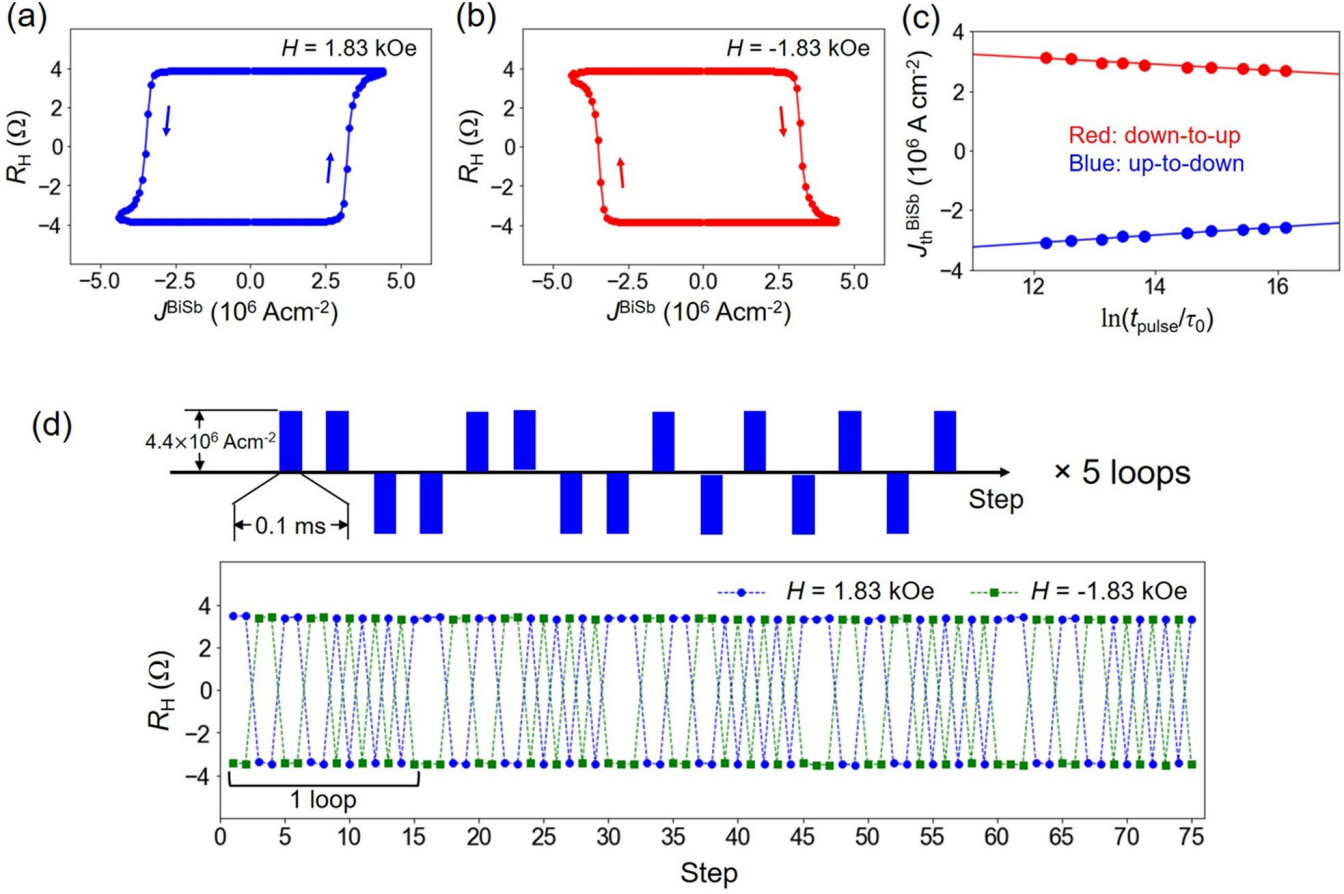
SOT magnetization switching by pulse currents. (**a**,**b**) Switching loop by 0.1 ms pulse currents under an in-plane magnetic field of *H* =  + 1.83 kOe and − 1.83 kOe, respectively. (**c**) Threshold current density $J_{{{\text{th}}}}^{{{\text{BiSb}}}}$ as a function of *t*_pulse_. (**d**) Robust SOT magnetization switching by 0.1 ms pulse current.

Next, we demonstrate ultrahigh efficient and robust SOT magnetization switching in the CoPt/BiSb multilayers. Figure 3 shows the SOT magnetization switching by DC currents with an applied external field along the *x* direction. We achieved Hall resistance switching whose amplitude is consistent with that of the Hall resistance loop shown in Fig. 1d, indicating full magnetization switching. The switching direction is reversed when the external magnetic field direction is reversed, which is consistent with the characteristic of SOT. Typical DC threshold switching current density $J_{{{\text{th}}}}^{{{\text{BiSb}}}}$ is 1.5 × 10^6^ Acm^−2^ at the bias field of 2.75 kOe. Note that thanks to the high electrical conductivity *σ* = 1.5 × 10^5^ Ω^−1^ m^−1^ of BiSb, the total current density including the shutting current in the CoPt is kept at 2.6 × 10^6^ Acm^−2^. Next, we performed SOT magnetization switching by pulse currents. Figure 4a,b show representative SOT switching loops by 0.1 ms pulse currents at + 1.83 kOe and − 1.83 kOe, respectively. Figure 4c plots $J_{{{\text{th}}}}^{{{\text{BiSb}}}}$ at various pulse width *t*_pulse_, and the theoretical fitting using the thermal activation model $J_{{{\text{th}}}}^{{{\text{BiSb}}}}$ = $J_{{0}}^{{{\text{BiSb}}}}$_×_$\left[ {1 - \frac{1}{\Delta }\ln \left( {\frac{{\tau_{{{\text{pulse}}}} }}{{\tau_{0} }}} \right)} \right]$^38^, where $J_{{0}}^{{{\text{BiSb}}}}$ is the zero-kelvin threshold switching current density, ∆ is the thermal stability factor, and 1/*τ*_0_ = 1 GHz (*τ*_0_ = 1 ns) is the attempt switching frequency. The fitting yields $J_{{0}}^{{{\text{BiSb}}}}$ = 4.6 × 10^6^ Acm^−2^ and Δ = 38. Because magnetization switching occurs by domain wall nucleation and domain wall motion, Δ reflects the energy barrier of the volume with size equal to the domain wall width, i.e. Δ should be considered as the energy barrier to nucleate a domain wall, rather than the energy barrier for coherently switching of the whole volume of the magnetic layer^39^. Therefore, Δ evaluated by this way is smaller than that should be expected for switching the whole volume of the magnetic layer. Nevertheless, the obtained Δ of CoPt is large enough to ensure that the total Δ in ferromagnetically (antiferromagnetically) coupled CoFeB/Ta(Ru)/CoPt free layer can exceeds 60 for 10 years thermal stability, while the switching current density remains the same^40^. Finally, we demonstrate robust SOT switching in the CoPt/BiSb junction. For this purpose, we applied a sequence of 150 pulses ($J_{{{\text{th}}}}^{{{\text{BiSb}}}}$ =  ± 4.4 × 10^6^ Acm^−2^, *t*_pulse_ = 0.1 ms) as shown in the top panel of Fig. 4c. The Hall resistance data recorded for a total of 150 pulses under ± 1.83 kOe are shown in the bottom panel in Fig. 4c. We observed a robust SOT switching with no change in the device characteristics, indicating that the BiSb topological insulator deposited by the sputtering technique has great potential for realistic SOT-MRAM.

## SOT performance benchmarking and future prospective

Table 1 summarizes *θ*_SH_, *σ*, the spin Hall conductivity *σ*_SH_ = (*ħ*/2*e*)*σθ*_SH_, and the SOT normalized power consumption *P*_n_ at room temperature of several heavy metals and TIs. Here, *θ*_SH_ of TIs are their best values reported in literature. For the calculation of the *P*_n_, we assumed bilayers of spin Hall material (thickness *t* = 6 nm for heavy metals and *t* = 10 nm for TIs) and CoFeB (thickness *t*_FM =_ 1.5 nm, conductivity *σ*_FM_ = 6 × 10^5^ Ω^−1^ m^−1^). Considering the shunting current in the ferromagnetic layer, the SOT power consumption is proportional to (*σt* + *σ*_FM_*t*_FM_)/*(σtθ*_SH_)^2^. One can see that not only *θ*_SH_ but also *σ* affect the SOT power consumption, a fact usually overlooked in literature. For example, while the sputtered Bi_*x*_Se_1-*x*_ has a much larger spin Hall angle (*θ*_SH_ = 18.6) than that (*θ*_SH_ = 3.5) of MBE-grown Bi_2_Se_3_, their power consumption is nearly the same, because Bi_*x*_Se_1-*x*_ has poorer crystal quality than Bi_2_Se_3_ and thus very low conductivity. Meanwhile, the sputtered BiSb thin film in this work shows both high *σ* = 1.5 × 10^5^ Ω^−1^ m^−1^ and large *θ*_SH_ = 10.7, which are optimal for both small switching current density and small switching power consumption^41^. Indeed, the switching power consumption for sputtered BiSb is 50 times smaller than that for sputtered Bi_*x*_Se_1-*x*_, and over 300 times smaller than that for W, which is the most used heavy metal in SOT-MRAM development. The small switching current density and switching power also help suppress failure of the spin Hall layer due to electromigration and Joule heating^42^. Our results demonstrate the feasibility of BiSb for not only ultralow power SOT-MRAM but also other SOT-based spintronic devices, such as race-track memories^43^ and spin Hall oscillators^44,45^.

**Table 1:** Spin Hall angle *θ*_SH_, electrical conductivity *σ*, spin Hall conductivity *σ*_SH_, and SOT normalized power consumption *P*_n_ of several heavy metals and topological insulators.

SOT materials	|*θ*_SH_|	*σ* (Ω^−1^ m^−1^)	|*σ*_SH_|[(*ħ*/2*e*) Ω^−1^ m^−1^]	*P*_n_
Ta	0.15	5.3 × 10^5^	8.0 × 10^4^	1
Pt	0.08	4.2 × 10^6^	3.4 × 10^5^	3.6 × 10^–1^
W	0.4	4.7 × 10^5^	1.9 × 10^5^	1.6 × 10^–1^
(Bi_0.07_Sb_0.93_)_2_Te_3_ (MBE)	2.5	1.8 × 10^4^	4.5 × 10^4^	3.0 × 10^–1^
Bi_2_Se_3_ (MBE)	3.5	5.7 × 10^4^	2.0 × 10^5^	2.1 × 10^–2^
Bi_x_Sei_1-x_ (sputtered)	18.6	7.8 × 10^3^	1.5 × 10^5^	2.6 × 10^–2^
Bi_0.85_Sb_0.15_ (sputtered)	10.7	1.5 × 10^5^	1.6 × 10^6^	5.2 × 10^–4^

Finally, we discuss about the remaining challenges for realization of ultralow power BiSb-based SOT-MRAM. One of material challenges is the large surface roughness of BiSb due to the unusually large crystal grain size comparing with the layer thickness. Furthermore, atomic interdiffusion between BiSb and the free magnetic layer during annealing process of the MTJ is also a challenge to be resolved. Thereby, a realistic pathway to integrate BiSb to SOT-MRAM is to fabricate full stack MTJs with PMA (p-MTJ) first, then deposit the BiSb layer on top of the MTJs at the last step after the annealing process, i.e. BiSb-on-MTJ structure. This will help avoid the interdiffusion and the BiSb surface roughness problems in the MTJ-on-BiSb structure.

## Method

### Material growth

We deposited multilayers of (0.4 nm Co / 0.4 nm Pt)_2_ / 10 nm Bi_0.85_Sb_0.15_ / 1 nm MgO / 1 nm Pt on *c*-plane sapphire substrates by DC (for Co, Pt, BiSb) and RF (for MgO) magnetron sputtering in a multi-cathode chamber. All layers are deposited by sputtering from their single targets using Ar plasma without breaking the vacuum at room temperature.

### Device fabrication

The samples were patterned into 90 μm-long × 25 μm-wide Hall bars by optical lithography and lift-off. A 45 nm-thick Pt were deposited as electrodes by DC magnetron sputtering, which reduces the effective length of the devices to 50 μm.

### SOT characterization

The samples were mounted inside a vacuumed cryostat equipped with an electromagnet. For the second harmonic measurements, a NF LI5650 lock-in amplifier was employed to detect the first and the second harmonic Hall voltages under sine wave excitation generated by a Keithley 6221 AC/DC current source. For the DC (pulse) current-induced SOT magnetization switching, a Keithley 2400 SourceMeter (6221 AC/DC current source) was used, and the Hall signal was measured by a Keithley 2182A NanoVoltmeter.

## Supplementary Information

Supplementary Information.

## Data Availability

The data that support this study results are available from the corresponding author upon reasonable request.

## References

[1] Zhao WS (2012). Failure and reliability analysis of STT-MRAM. Microelectron. Reliab..

[2] Liu L (2012). Spin–torque switching with the giant spin Hall effect of tantalum. Science.

[3] Garello K (2014). Ultrafast magnetization switching by spin-orbit torques. Appl. Phys. Lett..

[4] Miron IM (2011). Perpendicular switching of a single ferromagnetic layer induced by in-plane current injection. Nature.

[5] Liu L, Lee OJ, Gudmundsen TJ, Ralph DC, Buhrmam RA (2012). Current-induced switching of perpendicularly magnetized magnetic layers using spin torque from the spin Hall effect. Phys. Rev. Lett..

[6] Hao Q, Xiao G (2015). Giant Spin Hall effect and switching induced by spin-transfer torque in a W/Co40Fe40B20/MgO structure with perpendicular magnetic anisotropy. Phys. Rev. Appl..

[7] Garello K. *et al*. Manufacturable 300mm platform solution for Field-Free Switching SOT-MRAM. *2019 Symposium on VLSI Technology*, JFS4–5 (2019).

[8] Mellnik AR (2014). Spin-transfer torque generated by a topological insulator. Nature.

[9] Fan Y (2014). Magnetization switching through giant spin–orbit torque in a magnetically doped topological insulator heterostructure. Nat. Mater..

[10] Bernevig BA, Hughes TL, Zhang SC (2006). Quantum spin Hall effect and topological phase transition in HgTe quantum wells. Science.

[11] König M (2007). Quantum spin Hall insulator state in HgTe quantum wells. Science.

[12] Zhang H (2009). Topological insulators in Bi_2_Se_3_, Bi_2_Te_3_ and Sb_2_Te_3_ with a single Dirac cone on the surface. Nat. Phys..

[13] Hsieh D (2009). Observation of unconventional quantum spin textures in topological insulators. Science.

[14] Hirahara, T. *et al*. Topological metal at the surface of an ultrathin Bi_1− x_Sb_x_ alloy film. *Phys. Rev. B* **81**, 165422 (2010).

[15] Hai PN (2020). Spin Hall effect in topological insulators. J. Magn. Soc. Jpn..

[16] Wu H (2019). Room-temperature spin-orbit torque from topological surface states. Phys. Rev. Lett..

[17] Wang Yi (2017). Room temperature magnetization switching in topological insulator-ferromagnet heterostructures by spin-orbit torques. Nat. Commun..

[18] Han J (2017). Room-temperature spin-orbit torque switching induced by a topological insulator. Phys. Rev. Lett..

[19] Mahendra DC (2018). Room-temperature high spin–orbit torque due to quantum confinement in sputtered Bi_x_Se_(1–x)_ films. Nat. Mat..

[20] Teo JCY, Fu L, Kane CL (2008). Surface states and topological invariants in three-dimensional topological insulators: Application to Bi_1−x_Sb_x_. Phys. Rev. B.

[21] Ueda Y, Khang NHD, Yao K, Hai PN (2017). Epitaxial growth and characterization of Bi_1-x_Sb_x_ spin Hall thin films on GaAs(111)A substrates. Appl. Phys. Lett..

[22] Khang NHD, Ueda Y, Hai PN (2018). A conductive topological insulator with large spin Hall effect for ultralow power spin–orbit torque switching. Nat. Mater..

[23] Chi ZhD (2020). The spin Hall effect of Bi-Sb alloys driven by thermally excited Dirac-like electrons. Sci. Adv..

[24] Fan T, Khang NHD, Shirokura T, Huy HH, Hai PN (2021). Low power spin–orbit torque switching in sputtered BiSb topological insulator/perpendicularly magnetized CoPt/MgO multilayers on oxidized Si substrate. Appl. Phys. Lett..

[25] Roschewsky N (2019). Spin-orbit torque and nernst effect in Bi-Sb/Co heterostructures. Phys. Rev. B.

[26] Liu E, Swerts J, Couet S, Mertens S, Tomczak Y, Lin T, Spampinato V, Franquet A, Van Elshocht S, Kar G (2016). Co/Ni-CoFeB hybrid free layer stack materials for high density magnetic random access memory applications. Appl. Phys. Lett..

[27] Fan T, Tobah M, Shirokura T, Khang NHD, Hai PN (2020). Crystal growth and characterization of topological insulator BiSb thin films by sputtering deposition on sapphire substrates. Jpn. J. Appl. Phys..

[28] Gajek M (2012). Spin torque switching of 20 nm magnetic tunnel junctions with perpendicular anisotropy. Appl. Phys. Lett..

[29] Sato H (2014). Properties of magnetic tunnel junctions with a MgO/CoFeB/Ta/CoFeB/MgO recording structure down to junction diameter of 11 nm. Appl. Phys. Lett..

[30] Hayashi M, Kim J, Yamanouchi M, Ohno H (2014). Quantitative characterization of the spin-orbit torque using harmonic Hall voltage measurements. Phys. Rev. B.

[31] Kim J (2013). Layer thickness dependence of the current-induced effective field vector in Ta| CoFeB| MgO. Nat. Mater..

[32] Shao Q (2016). Strong Rashba-Edelstein effect-induced spin–orbit torques in monolayer transition metal dichalcogenide/ferromagnet bilayers. Nano Lett..

[33] Wu H (2019). Spin-orbit torque switching of a nearly compensated ferrimagnet by topological surface states. Adv. Mater..

[34] Khang NHD, Hai PN (2020). Spin–orbit torque as a method for field-free detection of in-plane magnetization switching. Appl. Phys. Lett..

[35] Shirokura T, Hai PN (2021). Angle resolved second harmonic technique for precise evaluation of spin orbit torque in strong perpendicular magnetic anisotropy systems. Appl. Phys. Lett..

[36] Jinnai B, Zhang CL, Kurenkov A, Bersweiler M, Sato H, Fukami S, Ohno H (2017). Spin-orbit torque induced magnetization switching in Co/Pt multilayers. Appl. Phys. Lett..

[37] Yasuda K (2017). Current-nonlinear Hall effect and spin-orbit torque magnetization switching in a magnetic topological insulator. Phys. Rev. Lett..

[38] Koch RH, Katine JA, Sun JZ (2004). Time-resolved reversal of spin-transfer switching in a nanomagnet. Phys. Rev. Lett..

[39] Sato H, Yamanouchi M, Miura K, Ikeda S, Koizumi R, Matsukura F, Ohno H (2012). CoFeB thickness dependence of thermal stability factor in CoFeB/MgO perpendicular magnetic tunnel junctions. IEEE Magn. Lett..

[40] Sato H, Yamanouchi M, Ikeda S, Fukami S, Matsukura F, Ohno H (2012). Perpendicular-anisotropy CoFeB-MgO magnetic tunnel junctions with a MgO/CoFeB/Ta/CoFeB/MgO recording structure. Appl. Phys. Lett..

[41] Li X, Lin ShJ, Mahendra Dc, Liao YCh, Yao ChY, Naeemi A, Tsai W, Wang SX (2020). Materials requirements of high-speed and low-power spin-orbit-torque magnetic random-access memory. IEEE J. Electron Devices Soc..

[42] Shiokawa Y, Komura E, Ishitani Y, Tsumita A, Suda K, Kakinuma Y, Sasaki T (2019). High write endurance up to 10^12^ cycles in a spin current-type magnetic memory array. AIP Adv..

[43] Ryu K-SS, Thomas L, Yang S-H, Parkin S (2013). Chiral spin torque at magnetic domain walls. Nat. Nanotech..

[44] Liu L, Pai C-F, Ralph DC, Buhrman RA (2012). Magnetic Oscillations Driven by the Spin Hall Effect in 3-Terminal Magnetic Tunnel Junction Devices. Phys. Rev. Lett..

[45] Shirokura T, Hai PN (2020). Bias-field-free spin Hall nano-oscillators with an out-of-plane precession mode. J. Appl. Phys..

