# Supplementary material for: Ultrahigh efficient spin orbit torque magnetization switching in fully sputtered topological insulator and ferromagnet multilayers
Source: Sci Rep. 2022 Feb 22;12:2998. doi: 10.1038/s41598-022-06779-3 (PMC8863830; doi:10.1038/s41598-022-06779-3)
Supplement: Supplementary file 1 — Supplementary Information. [file 41598_2022_6779_MOESM1_ESM.pdf]

Supplementary Information

Ultrahigh efficient spin orbit torque magnetization switching in fully sputtered topological insulator and ferromagnet multilayers

Tuo Fan[1], Nguyen Huynh Duy Khang[1,2], Soichiro Nakano[1], Pham Nam Hai[1,3,4]

[1]Department of Electrical and Electronic Engineering, Tokyo Institute of Technology, 2-12-1 Ookayama, Meguro, Tokyo 152-8550, Japan

[2]Department of Physics, Ho Chi Minh City University of Education, 280 An Duong Vuong Street, District 5, Ho Chi Minh City 738242, Vietnam

[3]Center for Spintronics Research Network (CSRN), The University of Tokyo, 7-3-1 Hongo, Bunkyo, Tokyo 113-8656, Japan

[4]CREST, Japan Science and Technology Agency, 4-1-8 Honcho, Kawaguchi, Saitama 332-0012, Japan

1. Criteria for spin Hall materials in SOT-MRAM

Here we discuss the criteria for spin Hall materials in SOT-MRAM, basing on simulation results by Xiang Li *et al.*, "Materials Requirements of High-Speed and Low-Power Spin-Orbit-Torque Magnetic Random-Access Memory", IEEE Journal of the Electron Devices Society, 8, 674 (2020), DOI: 10.1109/JEDS.2020.2984610. In this simulation, a 2 Transistor-1 MTJ architecture of SOT-MRAM cell with FIN-FET transistors was assumed. The resistance of the bottom electrode, top electrode, word line, source line, bit line, and the shunting current to the magnetic free layer were considered. Figure S1(a)(b) show the calculated switching current density and switching energy for various heavy metals, topological insulators, and semimetals, plotted against their sheet resistance $R_{\square}^{\mathrm{SOT}}$. The number behind the materials indicates their spin Hall conductivity σ_{SH} in unit of 10^5 $(\hbar/2e)\Omega^{-1}m^{-1}$. Four representative cases are indicated by the blue arrows: the heavy metal β−W [θ_{SH}=0.6, $\sigma = 4.3\times10^5$ $\Omega^{-1}m^{-1}$, $\sigma_{SH} = 2.6\times10^5$ $(\hbar/2e)\Omega^{-1}m^{-1}$] and three topological insulators: MBE-grown $Bi_{0.9}Sb_{0.1}$ [θ_{SH}=52, $\sigma = 2.5\times10^5$ $\Omega^{-1}m^{-1}$, $\sigma_{SH} = 130\times10^5$ $(\hbar/2e)\Omega^{-1}m^{-1}$], MBE-grown Bi_2Se_3 [θ_{SH}=3.5, $\sigma = 0.57\times10^5$ $\Omega^{-1}m^{-1}$, $\sigma_{SH} = 2.0\times10^5$ $(\hbar/2e)\Omega^{-1}m^{-1}$], and sputtered Bi_xSe_{1-x} [θ_{SH}=2.9, $\sigma = 0.48\times10^5$ $\Omega^{-1}m^{-1}$, $\sigma_{SH} = 1.39\times10^5$ $(\hbar/2e)\Omega^{-1}m^{-1}$]. One can see a general trend: materials that have high resistivity tend to have higher spin Hall angle, which leads to lower switching current. The Fig. S4(a) shows the lowest switching current for materials with $R_{\square}^{\mathrm{SOT}}$ between 300 – 3000 Ω (indicated by two vertical dashed lines). However, high resistivity of the SOT layer also means higher applied voltage, resulting in higher switching energy. This is indeed the case of MBE-grown Bi_2Se_3. Although Bi_2Se_3 requires lower switching current than β−W, its switching energy is higher than that of β−W. The sputtered Bi_xSe_{1-x} is even worse. Thus, $R_{\square}^{\mathrm{SOT}}$ between 100 – 700 Ω is required for low switching energy. Combing the two graphs,

$R_{\square}^{\mathrm{SOT}}$ between 300 – 700 Ω would yields the best performance in term of both switching current and switching energy. For a typical 10 nm-thick topological insulator layer, this benchmarking requires that $\sigma > 10^5\ \Omega^{-1}\mathrm{m}^{-1}$.

Next, given that the requirement $\sigma > 10^5\ \Omega^{-1}\mathrm{m}^{-1}$ is satisfied, the requirement of $\theta_{\mathrm{SH}} > 10$ comes from the fact that we need $\sigma_{\mathrm{SH}} > 10^6\ (\hbar/2e)\Omega^{-1}\mathrm{m}^{-1}$ to be at least one order of magnitude superior to $\sigma_{\mathrm{SH}} = 2.6\times10^5\ (\hbar/2e)\Omega^{-1}\mathrm{m}^{-1}$ of β–W. Furthermore, $\theta_{\mathrm{SH}} > 10$ is also needed for SOT-MRAM to be competitive to STT-MRAM. We recall that the switching current of SOT-MRAM is as large as 290 μA for 50 nm device using β–W [IEEE Symp. on VLSI Circuits, 194-195 (2019)]. Meanwhile, the typical switching current of 50 nm STT-MRAM is about 40 μA [Thomas et al., "Basic Principles, Challenges and Opportunities of STT-MRAM for Embedded Memory Applications", MSST 2017]. That mean θ_{SH} of around 5 is required for SOT-MRAM to have the same switching current as STT-MRAM. However, since SOT-MRAM requires two transistors while STT-MRAM requires only one, the foot print of SOT-MRAM will be twice that of STT-MRAM at the same switching current, thereby there is no merit for such a case. For SOT-MRAM to have the same or less foot print than STT-MRAM, the switching current should be smaller than STT-MRAM by at least a factor of two. Thus, θ_{SH} of at least 10 or more is required.

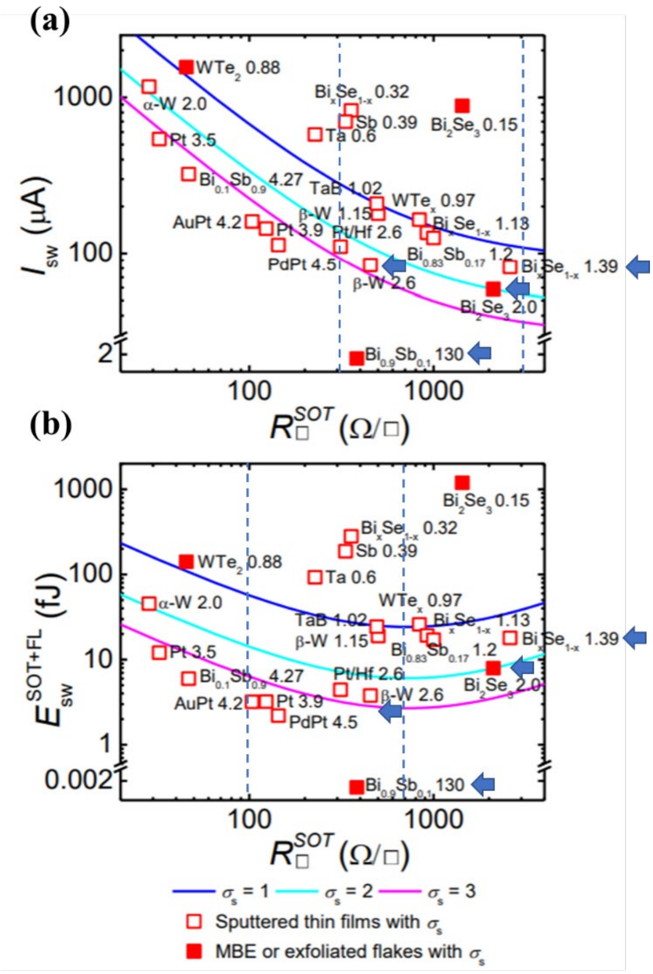

* σ_s unit is 1 x 10^5 $\hbar/2e(\Omega m)^{-1}$

Figure S1. (a) Switching current and **(b)** switching energy of SOT-MRAM using various materials as pure spin current source, plotted against their sheet resistance. [IEEE Journal of the Electron Devices Society, 8, 674 (2020), DOI: 10.1109/JEDS.2020.2984610]. Vertical lines indicate area with lowest switching current / switching energy.

2. Integration of topological insulator to SOT-MRAM

Fig. S2(a) show a representative MTJ stack developed with a high thermal stability factor of Δ ~ 80 at diameter of 50 nm. Typically, the perpendicular magnetic anisotropy (PMA) at a single CoFeB/MgO interface can yield only Δ ~ 40, which is not enough for STT-MRAM applications, thus a double interface MgO/CoFeB/Ta/CoFeB/MgO free layer was

proposed [Sato *et al.*, Appl. Phys. Lett. 101, 022414 (2012)], as shown in Fig. S2(a). Meanwhile, in Fig. S2(b), we show two possible scenarios for a BiSb-based SOT-MRAM. In the first scenario, BiSb is directly deposited on top of CoFeB. This scenario has some disadvantages and should be avoided. Since there is only one CoFeB/MgO interface, $\Delta \sim 40$ is small. Furthermore, we have shown that deposition of BiSb on a ferromagnetic layer can damage the ferromagnetic layer due to the large kinetic energy of Bi/Sb atoms, which reduces the effective spin Hall angle [Sci. Rep. 10, 12185 (2020)] and possibly reduce the TMR ratio. In the second scenario, we couple CoFeB to $(Co/Pt)_n$ multilayers ferromagnetically or antiferromagnetically with a middle Ta or Ru layer, then deposit BiSb on top of the $(Co/Pt)_n$ multilayers. This scenario has many advantages. First, the $(Co/Pt)_n$ multilayers add an extra Δ (~ 40 for $n = 2$ as demonstrated in this work) so that the total $\Delta \sim 80$ can be achieved. It has been shown that it is possible to increase Δ by this way without increasing the switching current density [Saito *et al.*, Appl. Phys. Lett. 101, 022414 (2012)]. Furthermore, we can increase the number of (Co/Pt) pairs to keep $\Delta > 60$ when the diameter is further reduced. Secondly, the $(Co/Pt)_n$ multilayers protect the CoFeB layer from diffusion of Bi/Sb atoms during BiSb deposition. Finally, we have demonstrated in this work that a large spin Hall angle larger than 10 can be achieved with the BiSb/$(Co/Pt)_n$ interface. Thus, the BiSb/$(Co/Pt)_n$/Ta(Ru)/CoFeB/MgO will be the better structure than BiSb/CoFeB/MgO for the free layer to utilize the giant spin Hall effect of BiSb for realistic SOT-MRAM.

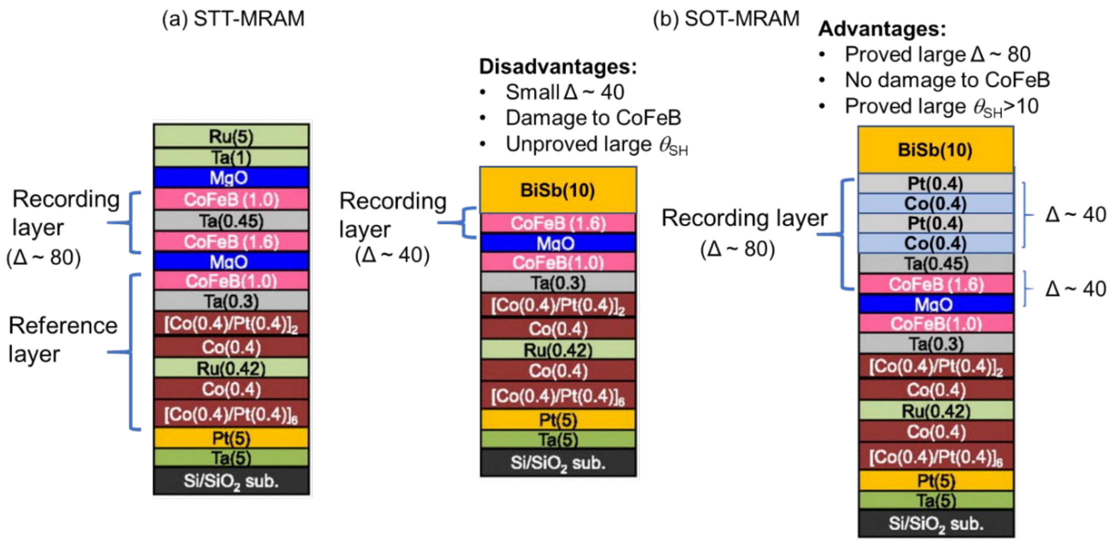

Figure S2. (a) STT-MRAM MTJ stack with a double interface MgO/CoFeB/Ta/CoFeB/MgO free layer (Ikeda *et al.*, 2014 IEDM, paper 32.2.1). **(b)** BiSb-based SOT-MRAM stack with CoFeB/BiSb (left) and CoFeB/Ta(Ru)/$(Co/Pt)_n$/BiSb (right) free layer. The right structure has many advantages over the left structure.

3. Structure analysis

We used X-ray diffraction (XRD) and transmission electron microscopy (TEM) to characterize the structure of the $(Co/Pt)_n$/BiSb multilayers. Figure S3(a) and S3(b) show a XRD θ-2θ spectrum and a cross-sectional TEM image of the $(Co/Pt)_n$/BiSb multilayers, which indicate that the deposited BiSb thin film has a dominant (110) orientation. Nevertheless, the BiSb thin film has a high conductivity of 1.5×10^5 $\Omega^{-1}m^{-1}$.

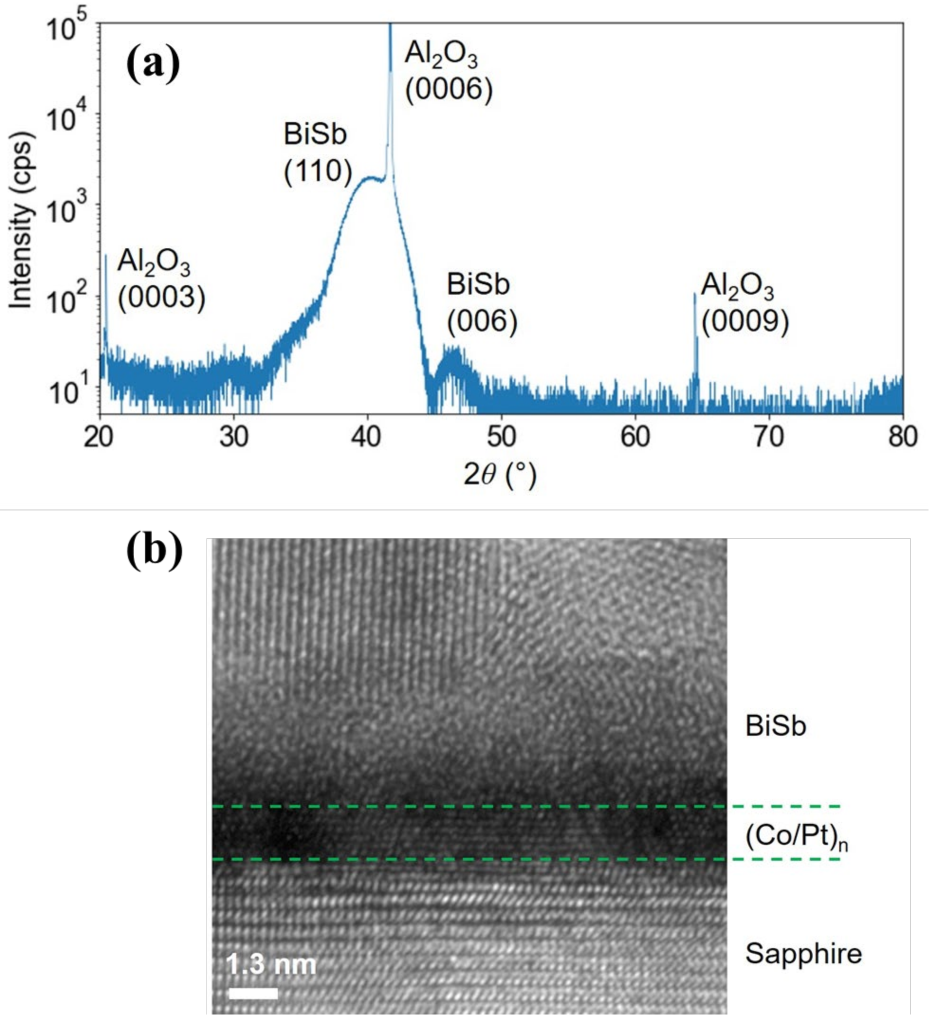

Figure S3. (a) XRD spectrum, and **(b)** cross-sectional TEM image of the $(Co/Pt)_n/BiSb$ multilayers deposited by magnetron sputtering on a sapphire substrate.

4. Perpendicular uniaxial anisotropy field after Hall bar fabrication

To estimate the perpendicular uniaxial anisotropy field of the (Co/Pt) multilayers after Hall bar fabrication, we measured the anomalous Hall resistance as function of the in-plane magnetic field. Figure S4 shows the anomalous Hall resistance as a function of the in-plane magnetic field. By fitting to $R_H = R_H(0)\sqrt{1-\left(\frac{H}{H_k}\right)^2}$, we obtained $H_k = 5.2$ kOe.

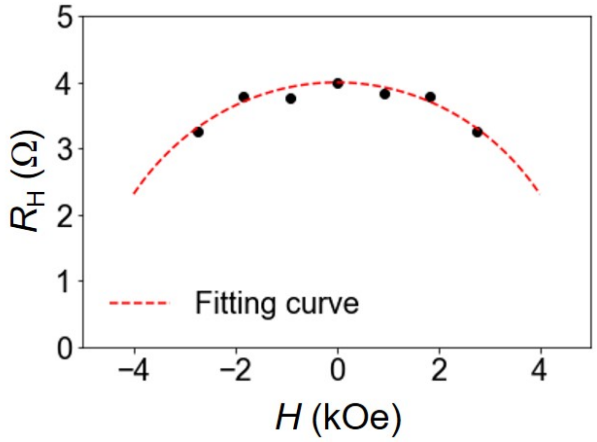

Figure S4. Anomalous Hall resistance in a Hall bar device as a function of the in-plane magnetic field.

5. Second harmonic Hall resistance data

Figure S5 shows the second harmonic Hall resistance data for estimation of the antidamping-like H_{AD} as a function of J^{BiSb}, which are summarized in Fig. 2(c) of the manuscript.

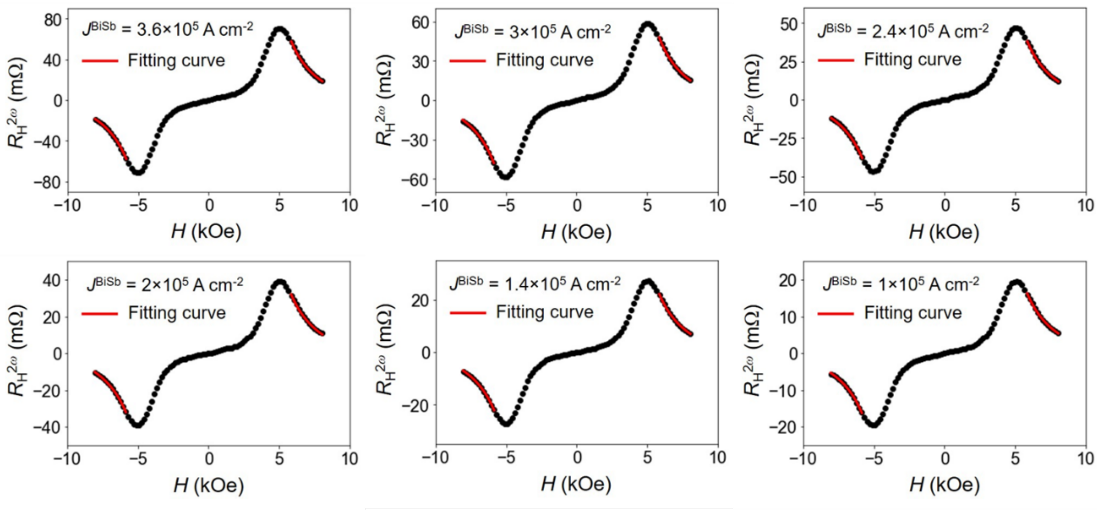

Figure S5. Second harmonic Hall resistance data for estimation of the antidamping-like H_{AD} as a function of J_{BiSb}.

6. Self spin-orbit torque in the (Co/Pt) multilayers

Recently, it was reported that (Co/Pt) multilayers can generate a “self” spin-orbit torque [Jinnai *et al.*, Appl. Phys. Lett. 111, 102402 (2017)]. To evaluate the contribution of the self-SOT effect in the (Co/Pt) multilayer, we performed control experiments on stand-alone $[Co(0.4)/Pt(0.4)]_2$ multilayers. We fabricated a 50 μm × 25 μm Hall bar similar to that in Fig. 1(b) of the manuscript. Figure S6(a) shows the anomalous Hall resistance of the $[Co(0.4)/Pt(0.4)]_2$ multilayers.

We first attempt to switch the magnetization of the $(Co/Pt)_2$ multilayers by the self-SOT effect. We applied the same current density to the $(Co/Pt)_2$ multilayers as that flowed into the $[Co/Pt]_2$ multilayers in the $[Co/Pt]_2$/BiSb heterostructure. In the first experiment shown in Fig. S6(b), we applied a DC current up to $\pm 1.38\times10^7$ Acm^{-2} under an in-plane bias field of 2.75 kOe. We observed no switching but Joule heating. Next, we attempt self-SOT switching by 1 ms and 0.1 ms pulse currents ramped up to $\pm 2.5\times10^7$ Acm^{-2} and $\pm 2.75\times10^7$ Acm^{-2}, respectively. Again, we observed no switching as shown in Fig. S6(c) and S6(d).

Next, we performed second harmonic measurements to evaluate the “self” spin-orbit torque and “self” spin Hall angle in the $[Co(0.4)/Pt(0.4)]_2$ multilayers. The results are shown in Fig. S7. By fitting the high field data in the $R_H^{2\omega}$- H_x curve (Figs. S7(a) – S7(e)) to the equation (1) in the manuscript, we obtained H_{AD} at each current density. Figure S7(f) shows H_{AD} as a function of J^{CoPt}, from which we evaluate that “self” the spin Hall angle of $[Co(0.4)/Pt(0.4)]_2$ is 0.26. This value is consistent with those observed in $Pt/(Co/Pt)_n$ by Jinnai *et al.* Appl. Phys. Lett. 111, 102402 (2017), which shows a maximum effective spin Hall angle of 0.30 for the underneath Pt layer. Considering the

current distribution in $[Co/Pt]_2$/BiSb heterostructure, we calculated that this "self" spin-orbit torque contributes to 13% of the total spin-orbit torque, and thereby not the main source of the observed SOT switching. By subtracting the contribution of the self-SOT from the raw data, we evaluate θ_{SH} of BiSb to be 10.7.

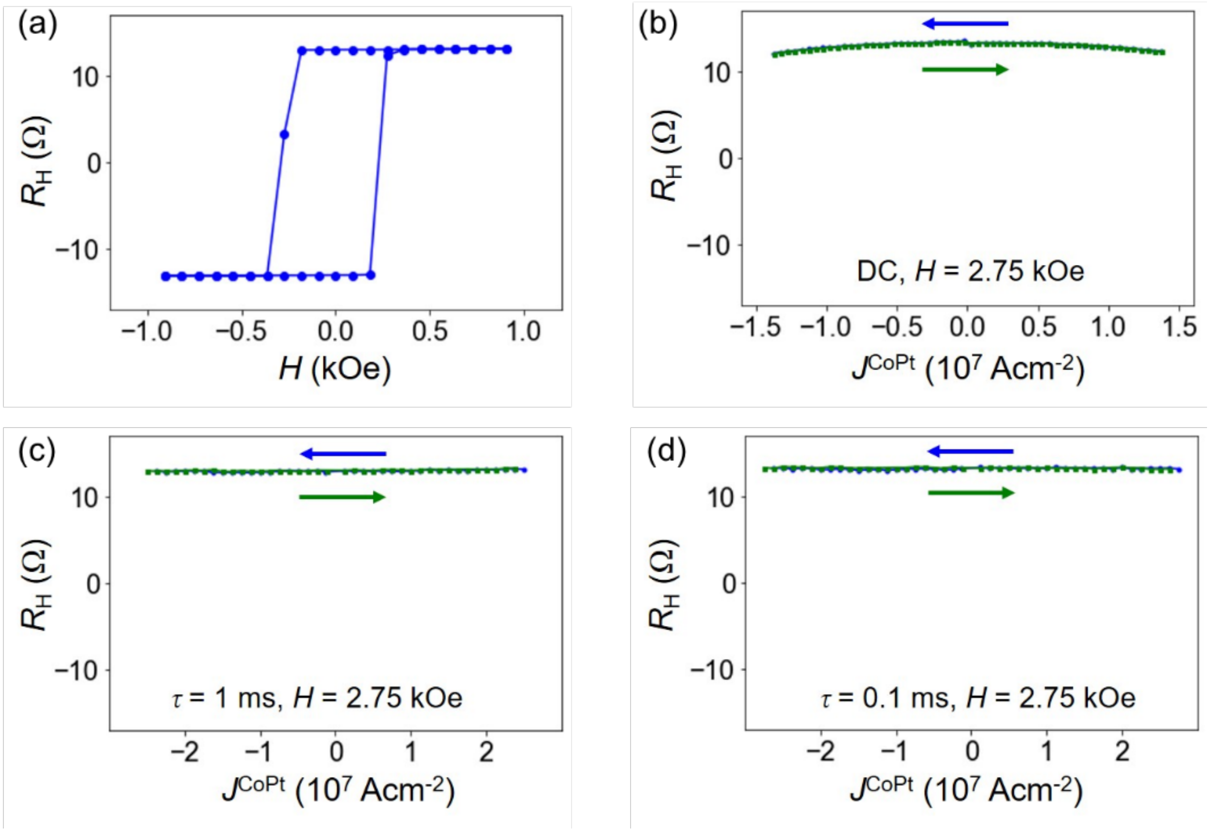

Figure S6. (a) Hall resistance of a $[Co/Pt]_2$ Hall bar device measured with a perpendicular magnetic field. Magnetization switching test for Co/Pt multilayers by **(b)** DC, **(c)** and **(d)** pulse current with pulse width of $\tau = 1$ ms and $\tau = 0.1$ ms, respectively.

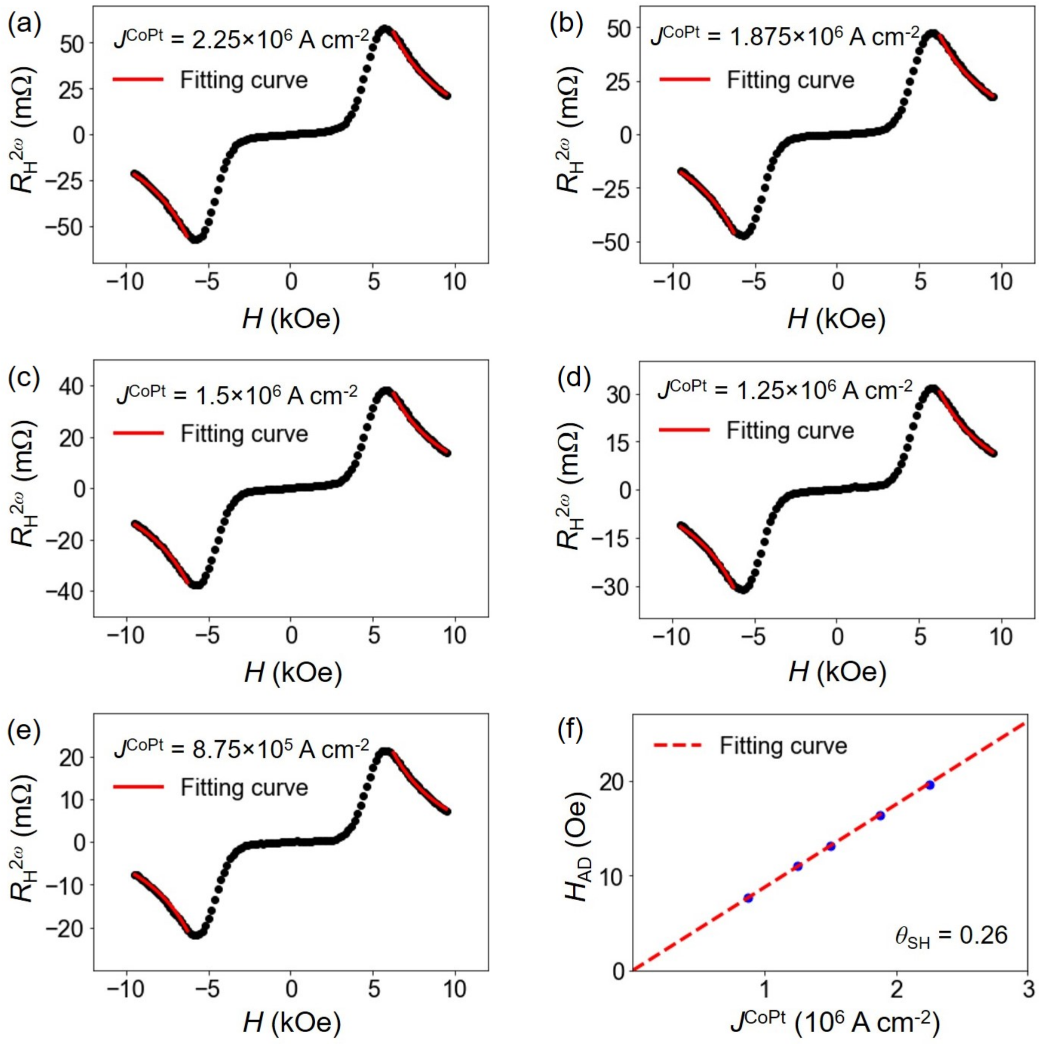

Figure S7. (a) – (e) 2nd harmonic Hall resistance as a function of in-plane magnetic field at various current densities J^{CoPt}. Solid lines are fitting curves using equation (1) in the manuscript. **(f)** H_{AD} as a function of J^{CoPt}.

7. On the asymmetric magnon scattering

Recently, it was observed that asymmetric magnon scattering in $(BiSb)_2Te_3/(CrBiSb)_2Te_3$ heterostructure [Phys. Rev. Lett. 119, 137204 (2017)] can results in a similar second harmonic signals to that SOT. Because the magnetic layer

$(CrBiSb)_2Te_3$ itself is a topological insulator, it has surface states with spin-momentum locking. The electron spins on the surface states of $(CrBiSb)_2Te_3$ are scattered by the magnons of Cr atoms in an asymmetric way due to spin-momentum locking. This is a very rare case and observed so far only in $(CrBiSb)_2Te_3$. Meanwhile, the magnetic layer in our study is just ferromagnetic metal $(Co/Pt)_n$ multilayers with no such topological surface states. Thus, asymmetric magnon scattering is unlikely in our case.

However, there might be a situation when the electron spins on the surface of BiSb are scattered by magnon in $(Co/Pt)_2$. Even if such a situation might be possible, we show three experimental evidences indicating that the magnon scattering is negligible in our $[Co/Pt]_2$/BiSb multilayers.

Evidence 1: Absence of the J^3 component

First, it is well known that magnon scattering rate is a non-linear function of the current density, and can be described by $aJ + bJ^3$. This is because the spin density generated by spin-momentum locking is proportional to J, while the magnon population increased by Joule heating is proportional to J^2. [Avci et al., Phys. Rev. Lett. 121, 087207 (2018); Khang *et al.* J. Appl. Phys. 126, 233903 (2019)]. It was observed that $bJ^3 >> aJ$ in these experiments. Thus, if the magnon scattering contribution was important in our experiment, $R_{xy}^{2\omega}$ would show $aJ + bJ^3$ dependence. In Fig. S8, we plot $R_{xy}^{2\omega}$ as a function as J^{CoPt} under various external in-plane magnetic fields. The $R_{xy}^{2\omega}$ – J^{CoPt} curves shows good linearity. **The absence of the J^3 component indicates magnon scattering is negligible in our experiment.**

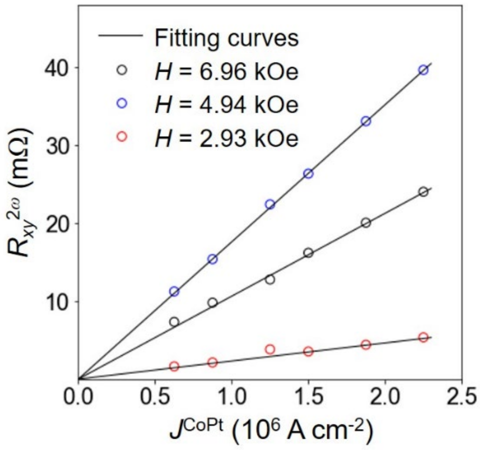

Figure S8. $R_{xy}^{2\omega}$ as a function as J^{CoPt}. Non-linearity due to magnon scattering was not observed.

Evidence 2: $\boldsymbol{R_{xy}^{2\omega}}$ **is not proportional to** $\boldsymbol{M_x}$.

Furthermore, $R_{xy}^{2\omega}$ originated from the asymmetric magnon scattering mechanism has a distinct feature that $R_{xy}^{2\omega}$ is proportional to the in-plane M_x component, as shown in Fig. S9(a) [replotted from Phys. Rev. Lett. 119, 137204 (2017)]. We examined whether this is the case in our BiSb/(Co/Pt)$_2$. In Fig. S9(b), we plot the normalized M_x as a function of the in-plane magnetic field H_x, calculated by $M_x/M = \sqrt{1-[R_H/R_H(0)]^2}$ from the anomalous Hall resistance data. If asymmetric magnon scattering dominated the second harmonic signal, we would see $R_{xy}^{2\omega} \propto M_x$. **However, the observed** $\boldsymbol{R_{xy}^{2\omega}}$ **in Fig. S9(c) is completely different from** $\boldsymbol{M_x}$ **in Fig. S9(b). Instead, the observed** $\boldsymbol{R_{xy}^{2\omega}}$ **is a text-book SOT curve:** $\boldsymbol{R_{xy}^{2\omega}}$ **sharply increases when** ***M*** **approaches the in-plane** ***x*****-direction, then drops at high fields following ~ 1/(**$\boldsymbol{H_x - H_u}$**).**

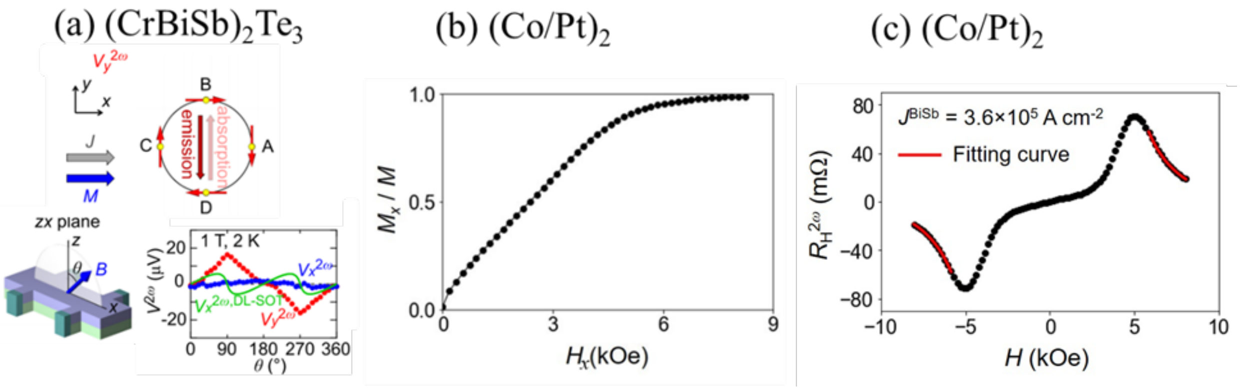

Figure S9. (a) Mechanism of asymmetric magnon scattering by spin-momentum locking and experimental evidence that shows $R_{xy}^{2\omega} \propto M_x$ in the magnetic topological insulator $(CrBiSb)_2Te_3$, replotted from PRL 119, 137204 (2017). **(b)** Normalized in-plane M_x component of $(Co/Pt)_2$ and **(c)** $R_{xy}^{2\omega}$ as a function of the in-plane magnetic field H_x in $(Co/Pt)_2$/BiSb multilayers. $R_{xy}^{2\omega} \propto M_x$ was not observed.

Evidence 3: $\boldsymbol{R_{xx}^{2\omega}(H//y)}$ is not observed.

According to the paper Phys. Rev. Lett. 119, 137204 (2017), **if the asymmetric magnon existed, one should observe $\boldsymbol{R_{xx}^{2\omega}(H//y)}$ = -3$\boldsymbol{R_{xy}^{2\omega}(H//x)}$.** Indeed, the authors in the Phys. Rev. Lett. 119, 137204 (2017) paper observed $R_{xx}^{2\omega}(H//y)$ with the same order of $R_{xy}^{2\omega}(H//x)$. To see whether this is the case, we measured $R_{xx}^{2\omega}(H//y)$ in $(Co/Pt)_2$/BiSb, i.e. when the magnetic field was applied in plane along the y direction. Figure S10 shows the $R_{xx}^{2\omega}$ measured with a magnetic applied in plane along y direction. **We observed no $\boldsymbol{R_{xx}^{2\omega}}$, which disagrees with the asymmetric magnon scattering mechanism.**

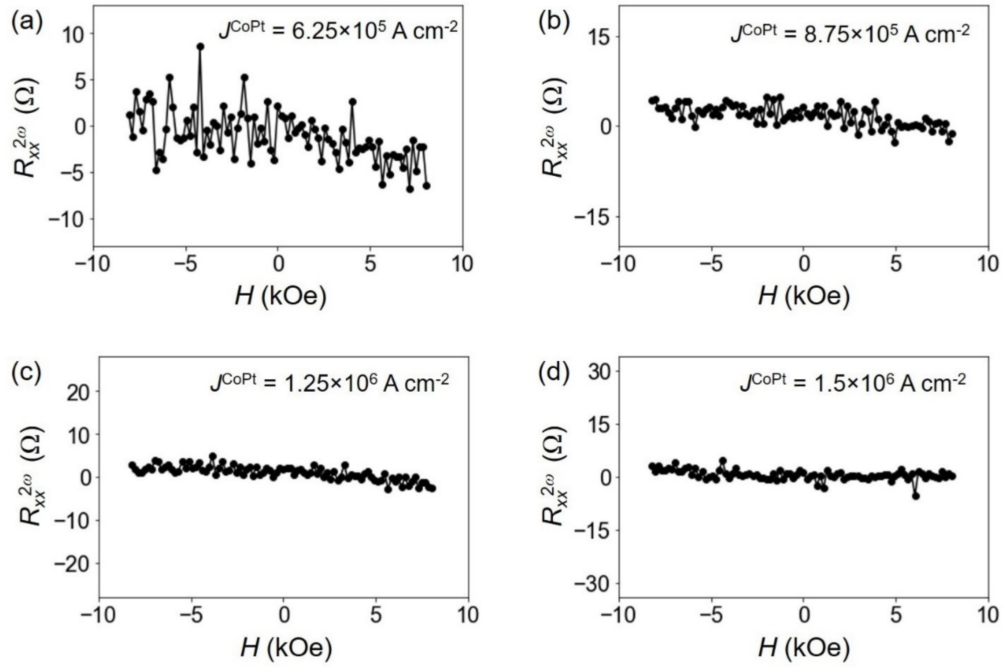

Figure S10. $R_{xx}^{2\omega}$ measured with an in-plane magnetic field applied along the *y* direction, at different current densities in $(Co/Pt)_2$ layers: **(a)** 6.25×10^5 Acm^{-2}, **(b)** 8.75×10^5 Acm^{-2}, **(c)** 1.25×10^6 Acm^{-2}, **(d)** 1.5×10^6 Acm^{-2}. No evidence of asymmetric magnon scattering was observed.

From these above observations, we conclude that asymmetric magnon scattering is negligible in $(Co/Pt)_2$/BiSb.
